# Acute Pancreatitis-Induced Euglycemic Diabetic Ketoacidosis

**DOI:** 10.7759/cureus.15949

**Published:** 2021-06-26

**Authors:** Arslan Chaudhry, Christopher Biggiani, Muhammad Afzal, Sohail Chaudhry, Yazan Vwich

**Affiliations:** 1 Internal Medicine, Saint Joseph's Regional Medical Center, Paterson, USA; 2 Endocrinology, Diabetes, and Metabolism, Saint Joseph's Regional Medical Center, Paterson, USA

**Keywords:** acute pancreatitis, euglycemic diabetic ketoacidosis, glp-1 agonist, sglt-2 inhibitor, type-2 diabetes mellitus

## Abstract

Glucagon-like peptide-1 receptor agonists (GLP-1 RAs) are a class of medications utilized for the treatment of diabetes mellitus by mechanisms promoting incretin release and insulin production. Although poorly understood, cases of acute pancreatitis have been observed in patients taking GLP-1 RAs. Sodium-glucose co-transporter-2 inhibitors (SGLT-2is) are another class of diabetic medications inhibiting renal glucose reabsorption which have been associated with rare cases of euglycemic ketoacidosis. Acute pancreatitis itself has been an observable cause of diabetic ketoacidosis, although typically in a hyperglycemia state. In this case report, we present a patient on SGLT-2is and GLP-1 RAs who developed acute pancreatitis, which may have precipitated euglycemic diabetic ketoacidosis (eu-DKA).

## Introduction

Type 2 diabetes mellitus (T2DM) is a chronic metabolic disease that is increasing in prevalence among the general population. The progression of diabetes and the need for supplementary glycemic control often requires a stepwise addition of glucose-lowering therapies, such as glucagon-like peptide-1 receptor agonists (GLP-1 RAs) and sodium-glucose co-transporter-2 inhibitors (SGLT-2is). GLP1-RAs regulate blood sugar by the release of incretins, stimulating insulin production in beta-pancreatic cells [1]. GLP-RAs have a black box warning regarding acute pancreatitis, which is suspected to be related to the release of incretins [1]. Dehydration is an early characteristic of acute pancreatitis which is believed to occur secondary to an increase in [Ca2+]i levels [2]. The presence of acute pancreatitis and dehydration may serve as predisposing factors for SGLT-2 inhibitor-associated diabetic ketoacidosis (DKA) [3]. SGLT-2is block the SGLT-2 protein, thereby inhibiting glucose reabsorption from the proximal renal tubule promoting glycosuria [4]. The reduced blood glucose levels decrease the secretion of endogenous insulin by pancreatic β-cells leading to increased hepatic ketogenesis [3]. In this case report, we present a 41-year-old male who developed acute pancreatitis and euglycemic diabetic ketoacidosis (eu-DKA) in the setting of concomitant GLP1-RAs and SGLT-2i use. It is important to note the implications of combination therapy of these two medications.

## Case presentation

A 41-year-old Syrian male with a past medical history of T2DM was presented to the emergency department with complaints of epigastric pain for a duration of one day. The pain was described as sharp, non-radiating and rated 10 out of 10. Symptoms were associated with right-sided chest discomfort, nausea, and two episodes of nonbilious and nonbloody vomiting. He denied any complaints of fevers or chills. He also denied a history of alcohol abuse, smoking, change in recent diet, travel, or sick contacts.

A list of his home medications included metformin 1000 mg PO BID, empagliflozin 12.5 mg PO BID, and semaglutide 1 mg subcutaneous injection once every week. At the time of presentation, vital signs were as follows: blood pressure, 123/78 mmHg; heart rate, 106 beats/min; respiratory rate, 20 breaths/min; temperature, 36.4°C; and BMI, 24.44. His physical examination was remarkable for mild epigastric tenderness. The initial metabolic panel showed elevation in the lipase levels >1300, mildly elevated triglycerides of 165, and positive serum acetone. However, blood sugar levels were noted to be within the normal range. An arterial blood gas (ABG) was also obtained which showed a pH of 7.21, pCO_2_ 16 mmHg, pO_2_ 107 mmHg, HCO_3_ 6.4 mmol/L. For further information, refer to the laboratory values below (Table 1).

**Table 1:** Patient's laboratory values CRP: C-reactive protein; MCV: mean corpuscular volume

Laboratory Parameters	Patient Values	Normal Range
Sodium- mEq/L	135	135-145
Potassium- mEq/L	4.4	3.5-5.0
Chloride- mEq/L	96	98-107
Bicarbonate- mEq/L	12	21-31
Glucose- mg/dL	119	70-110
Calcium- mg/dL	9.6	8.6-10.3
Phosphorus- mEq/L	2.6	2.5-5.0
Magnesium- mEq/L	1.6	1.7-2.5
Blood urine nitrogen- mg/dL	15	7-23
Serum creatinine- mg/dL	1.06	0.6-1.3
Bilirubin total- mg/dL	0.4	0.3-1.1
Protein total- g/dL	8.0	6.4-8.4
Albumin- g/dL	5.3	3.5-5.7
Alkaline phosphatase- units/L	57	34-104
Aspartate aminotransferase- units/L	22	13-39
Alanine aminotransferase-units/L	19	7-52
Total cholesterol- mg/dL	155	<199
Triglycerides- mg/dL	173	<149
Hemoglobin A1C- %	6.8	4-6
Lipase- units/L	1313	11-82
CRP- mg/L	27.7	≤9.9
Lactic acid- mmol/L	0.7	0.5-2.2
White blood cell count, x 10^3^/mm^3^	9.3	4.5-11.0
Hemoglobin- g/dL	16.6	13.5-17.5
Hematocrit- %	50.8	41.0-53.0
MCV- fL	87.9	80-100
Platelet- k/mm^3^	241	140-440

**Figure 1:**
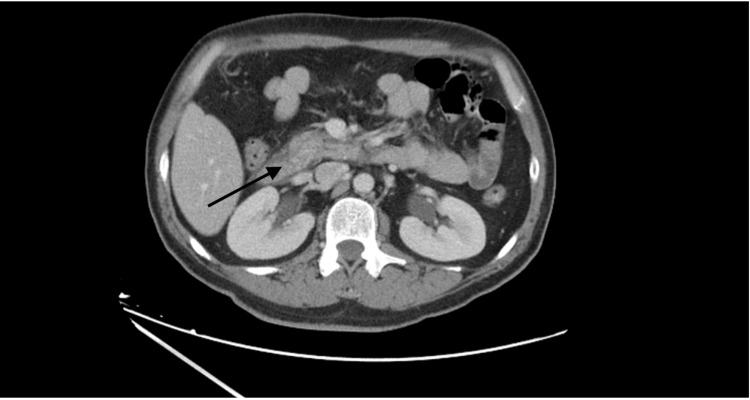
CT of the abdomen revealed pancreatitis of the head of the pancreas with adjacent duodenitis.

The ultrasound (US) of the abdomen demonstrated a 7 mm echogenic nodule suggestive of a gallbladder wall polyp. Further imaging was performed with a CT scan of the abdomen and pelvis with contrast which revealed pancreatitis of the head of the pancreas with adjacent duodenitis (Figure 1).

The patient was initially admitted to the medical floors for acute pancreatitis and started on aggressive IV hydration. A repeat ABG (pH of 7.17, pCO_2_ 17 mmHg, pO_2_ 68 mmHg, HCO_3_ 6.2) performed in the evening showed severe metabolic acidosis with an anion gap of 27, presence of urine ketones, normal blood glucose, and lactic acidosis. Due to the worsening of acidosis, the patient was transferred to the medical ICU for further management of euglycemic ketoacidosis and acute pancreatitis. He was continued on intravenous fluids, started on a bicarbonate drip and an insulin drip at a rate of 0.1-0.3 units/kg. Upon improvement of the anion gap, he was transitioned to subcutaneous insulin and transferred to the medical floors. The patient’s home medications, empagliflozin, and semaglutide were held during the hospital course due to the possible involvement in causing euglycemic ketoacidosis and acute pancreatitis, respectively.. The patient was discharged home after one week on metformin 500 mg twice daily and glimepiride 5 mg once daily with instructions to follow-up with his primary care physician.

## Discussion

Eu-DKA is an uncommon form of DKA that is characterized by euglycemia (blood sugars <250 mg/dL) in the presence of metabolic acidosis (arterial pH <7.3 and serum bicarbonate <18 mEq/L) [5]. Although Eu-DKA is becoming more prevalent, it is still a rare event associated with the use of SGLT-2is [4]. SGLT-2is are a class of drugs that work by inhibiting the SGLT2 protein, which decreases the concentrations of plasma glucose by inhibiting proximal tubular reabsorption of glucose in the kidney [6]. The proposed mechanism of SGLT-2i-induced euDKA suggests that the lowered glucose levels lead to decreased endogenous insulin production by the pancreatic β-cells [3]. This lower dose of insulin may be insufficient to suppress lipolysis and hepatic ketogenesis. It also stimulates the pancreatic α-cells, promoting glucagon secretion [3]. In addition, SGLT-2 inhibitors increase the reabsorption of acetoacetate in the renal tubules, which increases the blood level of ketone bodies [3]. Some predisposing risk factors for SGLT-2 inhibitor-associated DKA include latent autoimmune diabetes in adults, major surgeries, insulin noncompliance, acute medical illness, low-carbohydrate diet, acute pancreatitis, and dehydration [3]. In our case report, the 41 year-old-patient was on combination therapy of GLP-1 RA and SGLT-2 inhibitor. He presented with mild epigastric tenderness, elevated lipase >1300 U/L, and CT scan findings characteristic of acute pancreatitis. After ruling out the more common causes for acute pancreatitis, it was concluded that he had developed acute pancreatitis secondary to the use of GLP-1 RA. GLP-1 RAs are known to regulate blood sugar by the release of incretins which have been related to acute pancreatitis. The incretins (i.e., glucagon-like peptide 1 and glucose-dependent insulinotropic polypeptide) are intestinal hormones that regulate the postprandial production of insulin and glucagon by the pancreas [7]. Although there has been limited evidence related to this phenomenon, it is something that can not be excluded and must be considered a risk factor while assessing for all possible etiologies. The FDA undertook comprehensive evaluations of a safety signal arising from postmarketing reports of pancreatitis and pancreatic cancer in patients using incretin-based drugs [7]. Two clinical trials known as the SAVOR and EXAMINE were conducted which involved 16,492 and 5380 patients, respectively. Both trials were randomized, double-blinded, placebo-controlled. Reported rates of acute pancreatitis in the SAVOR and EXAMINE trials were low, with similar rates of events in the drug and placebo groups (22 and 16, respectively, in SAVOR; 12 and 8, respectively, in EXAMINE) [7]. Despite the limited likelihood, the FDA determined pancreatitis to be considered a risk associated with these drugs until more data becomes available [7]. Dehydration is an early characteristic of acute pancreatitis which is believed to occur secondary to an increase in [Ca2+]i levels [2]. We hypothesized that in our case, the euDKA was precipitated by acute pancreatitis and dehydration in the setting of combination therapy with SGLT-2i and GLP1-RA use.

## Conclusions

Eu-DKA is an uncommon cause of DKA related to insulin deficiency that is becoming more prevalent with the use of SGLT-2is. There are various risk factors that may precipitate SGLT-2i-induced DKA. Recognizing these factors plays a critical role in identifying the patient population at risk of developing DKA. There is limited data regarding acute pancreatitis precipitating eu-DKA in the setting of concomitant use of SGLT-2i and GLP1-RA which requires further studies to establish a correlation. 
